# Purification and characterization of polygalacturonase from *Aspergillus fumigatus* MTCC 2584 and elucidating its application in retting of *Crotalaria juncea* fiber

**DOI:** 10.1007/s13205-016-0517-4

**Published:** 2016-09-22

**Authors:** Gautam Anand, Sangeeta Yadav, Dinesh Yadav

**Affiliations:** Department of Biotechnology, D.D.U Gorakhpur University, Gorakhpur, UP 273 009 India

**Keywords:** Polygalacturonase, *Aspergillus fumigatus* MTCC 2584, Endo-PG, Retting, *Crotalaria juncea* fiber, Purification

## Abstract

Polygalacturonases represents an important member of pectinases group of enzymes with diverse industrial applications and is widely distributed among fungi, bacteria, yeasts, plants and some plant parasitic nematodes. An endo-polygalacturonase from a new fungal source *Aspergillus fumigatus* MTCC 2584 was produced under solid-state fermentation conditions and was purified simply by acetone precipitation and gel-filtration chromatography technique. The approximate molecular weight of the purified PG was found to be 43.0 kDa as revealed by SDS-PAGE. The pH optimum of the purified enzyme was found to be 10.0 and was stable in the pH range of 7–10. The optimum temperature of purified PG was found to be 30 °C. The *Km* and *Kcat* of the purified enzyme were 2.4 mg/ml and 44 s^−1^, respectively, and the metal ions Cu^2+^ and K^+^ were found to enhance the enzyme activity while Ag^+^, Ca^2+^ and Hg^2+^ were inhibitory in nature. Based on its alkaline nature, the potential of purified PG in retting of natural fiber *Crotalaria juncea* was elucidated in the absence of EDTA. This is probably the first report of alkaline PG from *Aspergillus fumigatus*.

## Introduction

Polygalacturonases (PGs) (EC 3.2.1.15) are important member of pectinases which catalyze the hydrolysis of α-1,4-D-galacturonic acid linkages in smooth region of pectin. Depending on their mode of hydrolysis of the substrate, PGs are classified as endo-PGs (E.C. 3.2.1.15) and exo-PGs (EC 3.2.1.67). The endo-PGs cleave the α-1,4-D-galacturonic acid linkages randomly, whereas exo-PGs cleave the linkage from non-reducing end (Anand et al. 2014). Endo-PGs are generally found in extracellular environment and exo-PGs in the periplasm. This localization is in coherence with the substrate accumulation in each environment, endo-PG would degrade extracellular polymerized forms of pectin present within the plant cell wall and exo-PGs would produce small oligogalacturonides and monogalacturonic acid from pectic fragments that accumulate in periplasm for intracellular transport (Abbott and Boraston 2008).

Polygalacturonases are widely distributed among fungi, bacteria, yeasts, plants and some plant parasitic nematodes (Niture 2008). The genera *Aspergillus* is an important source of PGs. Several endo-PGs and exo-PGs have been purified and characterized from different species of *Aspergillus* like *Aspergillus niger* (Parenicova et al. 1998; Zhou et al. 2015), *A. awamori* (Nagai et al. 2000), *A. carbonarius* (Nakkeeran et al. 2011), *A. tubingensis* (Kester et al. 1996), *A. niger* (Sakamoto et al. 2002), *A. sojae* (Dogan and Tari 2008) and *Aspergillus sojae* (Buyukkileci et al. 2014).

PGs being of great industrial importance, knowledge of their biochemical properties is essential for their utilization in relevant industries. The pH optima of purified PGs determines its possible application like fruit juice clarification, retting of natural fibers etc. It has been observed that most of the fungal PGs have pH optima 3–6 (Patil et al. 2012; Yadav et al. 2012; Kant et al. 2013; Martins et al. 2013; Castruita-Domínguez et al. 2014; Ortega et al. 2014; Chen et al. 2014; Zhou et al. 2015; Zaslona and Trusek-Holownia 2015; Pan et al. 2015).

Most of the fungal PGs show temperature optima between 35 and 60 °C, though few thermophilic fungal strains like *Aspergillus sojae*, *Paecilomyces variotii, Thermoascus aurantiacus* with temperature optima in the range of 60–70 °C have been reported (Dogan and Tari 2008; de Lima Damasio et al. 2010; Martins et al. 2007, 2013).

Alkaline polygalacturonases have been reported from bacterial strains, *Bacillus* in particular (Kobayashi et al. 2001), and alkaline PG having application in retting of natural fibers is a rare finding. Keeping the above points in view, authors have reported purification and characterization of an alkaline PG from fungal strain *Aspergillus fumigatus* MTCC 2584 under solid-state fermentation conditions and elucidated its application in retting of *Crotalaria juncea* fibers. Thus, it could be a potential enzyme for textile or paper industries.

## Materials and methods

### Chemicals

Polygalacturonic acid (PGA) and Sephadex G-100 were purchased from Sigma Chemical Company (St. Louis, MO, USA). Rests of the chemicals were procured either from Merck (Navi Mumbai, India) or S.D. Fine (Mumbai, India) and were used without further purification.

### Microorganism and culture condition

The fungal strain *Aspergillus fumigatus* MTCC 2584 was procured from Microbial Type Culture Collection and Gene Bank, Institute of Microbial Technology, Chandigarh (India) and screened for pectinase production by plate assay method (Molina et al. 2001). The culture was maintained by cultivation on Czapek-Dox agar slants at 26 °C.

### Solid-state fermentation

The enzyme was produced by solid-state fermentation. The production medium consisted of wheat bran 4.5 g, tea extract 0.5 g and 5 ml salt solution. The composition of salt solution was (4 g/L each of K_2_HPO_4_, KH_2_PO_4_, and NH_4_SO_4_). The organism was grown on Czapek-Dox agar slants. The inoculum was prepared by suspending the spores of the slants in sterile distilled water and counted using a hemocytometer. Ten 250 ml Erlenmeyer flasks containing solid medium were inoculated with 1 ml spore suspension (5 × 10^6^ spores/ml) in each flask; the flasks were kept at 26 °C in a biological oxygen demand (BOD) incubator. Maximum production of the enzyme occurred on 5th day of inoculation.

### Enzyme extraction

On fifth day, 15 ml chilled distilled water was added to each flask and the resulting mixture was homogenized by a glass rod. The homogenate was extracted through four layers of cheese cloth and the filtrate was centrifuged at 14,000 g for 20 min and clear supernatant was obtained that was used as a crude enzyme for further purification.

### PG assay

Enzyme activity of PG was assayed by determining the liberated reducing end products by standard method (Miller 1959). The reaction solution (2 mL) consisted of 0.5 mL of 1 % PGA, 1.4 mL 100 mM glycine-NaOH buffer (pH 10.0) and 0.1 mL enzyme solution. It was incubated for 20 min at 37 °C in a water bath. Three mL of dinitrosalicyclic acid (DNSA) reagent was added and volume was made 6 mL by addition of 1 mL distilled water. The solution was boiled for 10 min in a water bath, cooled and absorbance was read at 575 nm in a colorimeter. A control was simultaneously prepared taking thermally denatured enzyme. The concentration of the product (galacturonic acid) was determined with the help of a calibration curve. One unit of PG activity is defined as the amount of enzyme that liberates 1 μmol of galacturonic acid per min under the assay conditions. All experiments were performed in triplicates and were found to have standard deviation of less than 5 %.

### Enzyme purification

Chilled acetone was added slowly up to 60 % saturation with gentle stirring at 4 °C to the crude enzyme extract. The treated crude enzyme solution was allowed to stand overnight in the refrigerator, and centrifuged at 10,000 rpm for 15 min. The supernatant was discarded and pellet was dissolved in 3 mL of cold distilled water. The concentrated enzyme after acetone precipitation was loaded on a Sephadex G-100 column (1.0 × 30.0 cm), pre-equilibrated with 100 mM citrate phosphate buffer (pH 7.0). The flow rate was maintained at 12.0 ml/h and fractions were collected and analyzed for protein and polygalacturonase activity. The active fractions were pooled, assayed and stored in deep fridge at −20 °C. The purity of the enzyme was checked by sodium dodecyl sulfate-polyacrylamide gel electrophoresis (SDS–PAGE) in 10 % acrylamide gel according to Laemmli (1970).

### Biochemical characterization of purified polygalacturonase

The pH optimum was determined by measuring steady-state velocity using 0.5 g % PGA in the buffered reaction solution using different buffers at 100 mM in the pH range 1.0–12.0. The different buffers used were: hydrochloric acid–potassium chloride (pH 1.0–2.0), citrate–phosphate (pH 3.0–7.0), sodium phosphate (pH 8.0), glycine–sodium hydroxide (pH 9.0–10.0) and sodium phosphate–sodium hydroxide (pH 11.0–12.0). The pH stability of the enzyme was studied by exposing the enzyme to buffers of different pH for 24 h at 4 °C. The activities were assayed by the method described in enzyme assay section and plotted in the form of relative activity versus pH.

The optimum temperature for the enzyme activity was determined by assaying the activity of the enzyme at different temperatures in the range 10–100 °C and plotting a graph of the enzyme activity versus temperature of the reaction solutions. Thermal stability of the enzyme was tested by incubating enzyme aliquots at a particular temperature (10–100 °C) for 2 h and their activities were assayed using the standard assay method.

The *Km* and *Kcat* values of the purified enzyme were determined by measuring steady-state velocities of the enzyme-catalyzed reaction at different concentrations of citrus PGA (0.05–0.6 g % w/w) in 100 mM glycine–NaOH buffer (pH 10.0) at 30 °C and drawing double reciprocal plot (Engel 1977). Calculations were made using linear regression analysis of the data points of the double reciprocal plot.

### Effect of metal ions and protein inhibitors

The effects of metal ions, such as Ca^2+^, Mn^2+^, Co^2+^, Cu^2+^, Zn^2+^, Hg^2+^, K^+^, Na^+^, Ag^+^ and protein inhibitors like potassium permanganate, potassium ferrocyanide and ethylenediaminetetraacetic acid (EDTA), were studied by measuring the steady-state velocity in the reaction solutions containing 1 mM of the metal ions or protein inhibitors and comparing it with the value in the absence of these ions or inhibitors.

### Mode of action of purified polygalacturonase

To decide whether the purified PG is an exo- or endo-PG, a reaction solution containing 0.5 mL of 1 % PGA in distilled water and 1.4 mL of 100 mM glycine–NaOH buffer (pH 10.0) was added in a test tube. The test tube was incubated in a water bath at 30 °C and was allowed to stand to maintain the temperature for 20 min; 0.5 unit of the purified enzyme was added. 3 μL aliquots of the reaction solution were withdrawn at 15, 30 and 45 min and spots of these solutions were made on thin layer chromatography (TLC) plate coated with silica gel. Spots of monogalacturonic acid as well as PGA were also made. The TLC plate was kept in a jar containing a solution of butanol, water and acetic acid in the volume ratio 5:3:2 as mobile phase. The TLC plates were air dried, and sprayed with 0.2 % (w/v) orcinol in methanol and 10 % sulfuric acid. The plate was air dried and kept in an oven at 80 °C for 10 min. The spots were photographed.

### Retting of *Crotalaria juncea* fiber by purified PG

The retting of *Crotalaria juncea* natural fiber was carried out by the reported method (Zhang et al. 2005) with minor modifications. Approximately, 5.0 cm long sticks were kept in three test tubes. In each test tube, 10 mL of 100 mM glycine–NaOH buffer (pH 10.0) was added. One test tube was made control designated as ‘A’ and contained deactivated enzyme. The other test tubes were designated as ‘B’ containing purified enzyme with EDTA and ‘C’ containing purified enzyme without EDTA. All the test tubes were incubated in a water bath at 37 °C for 24 h. After 24 h, the sticks were shaken vigorously each with 10 mL hot water for 1 min, hot water was poured off and the resulting sticks were photographed.

## Results and discussion

### Purification of polygalacturonase

The purification of PG produced by *Aspergillus fumigatus* MTCC 2584 was performed by acetone precipitation and gel-filtration column chromatography. The purification chart is shown in Table 1.

**Table 1:** Purification chart of polygalacturonase produced from *Aspergillus fumigatus* MTCC 2584

Fraction	Total activity (U)	Total protein (mg)	Specific activity (U/mg)	Purification fold	(%) Yield
Crude	275	130	2.11	–	–
Acetone (0–60 %)	11.91	1.80	6.61	3.13	4.33
Sephadex G-100	8.18	0.21	38.9	18.43	2.98

The crude enzyme harvested on fifth day of fermentation and the clear culture filtrate was centrifuged and subjected to acetone precipitation (0–60 % saturation). The precipitate obtained after the acetone precipitation revealed 3.13-fold purification with 4.33 % yield and 6.61 U/mg specific activity (Table 1). The concentrated enzyme after acetone precipitation was loaded on a Sephadex G-100 column (1.0 × 30.0 cm), pre-equilibrated with 100 mM citrate phosphate buffer (pH 7.0). The flow rate was maintained at 12.0 ml/h and fractions were collected and analyzed for protein and PG activity. The gel-filtration chromatography resulted in an 18.43-fold purification with specific activity of 38.9 U/mg protein and 2.98 % yield (Table 1).

**Fig. 1:**
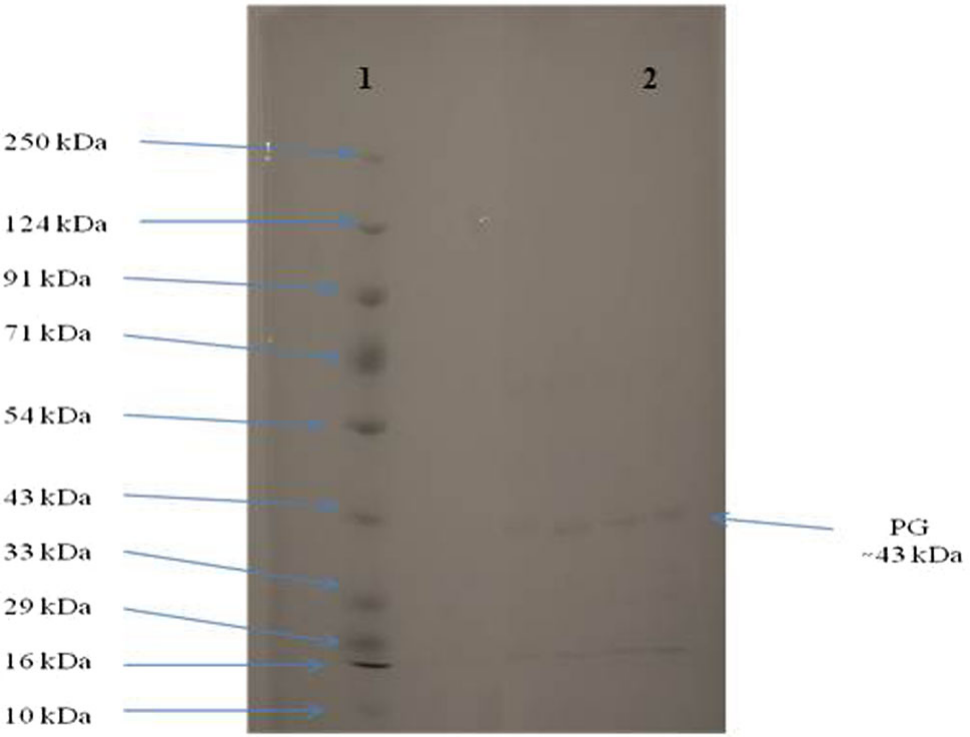
10 % SDS-PAGE of purified polygalacturonase (PG) from *A. fumigatus* MTCC 2584. *Lane 1* Protein molecular weight marker, *Lane 2* Purified PG

The purified polygalacturonase was confirmed for electrophoretic homogeneity by SDS-PAGE. The single band corresponding to a relative molecular mass of approximately 43.0 kDa was observed **(**Fig. 1). An endo-PG of 41.0 kDa has been reported from *Aspergillus awamori* (Nagai et al. 2000).

### Effect of pH on the activity and stability of PG

**Fig. 2:**
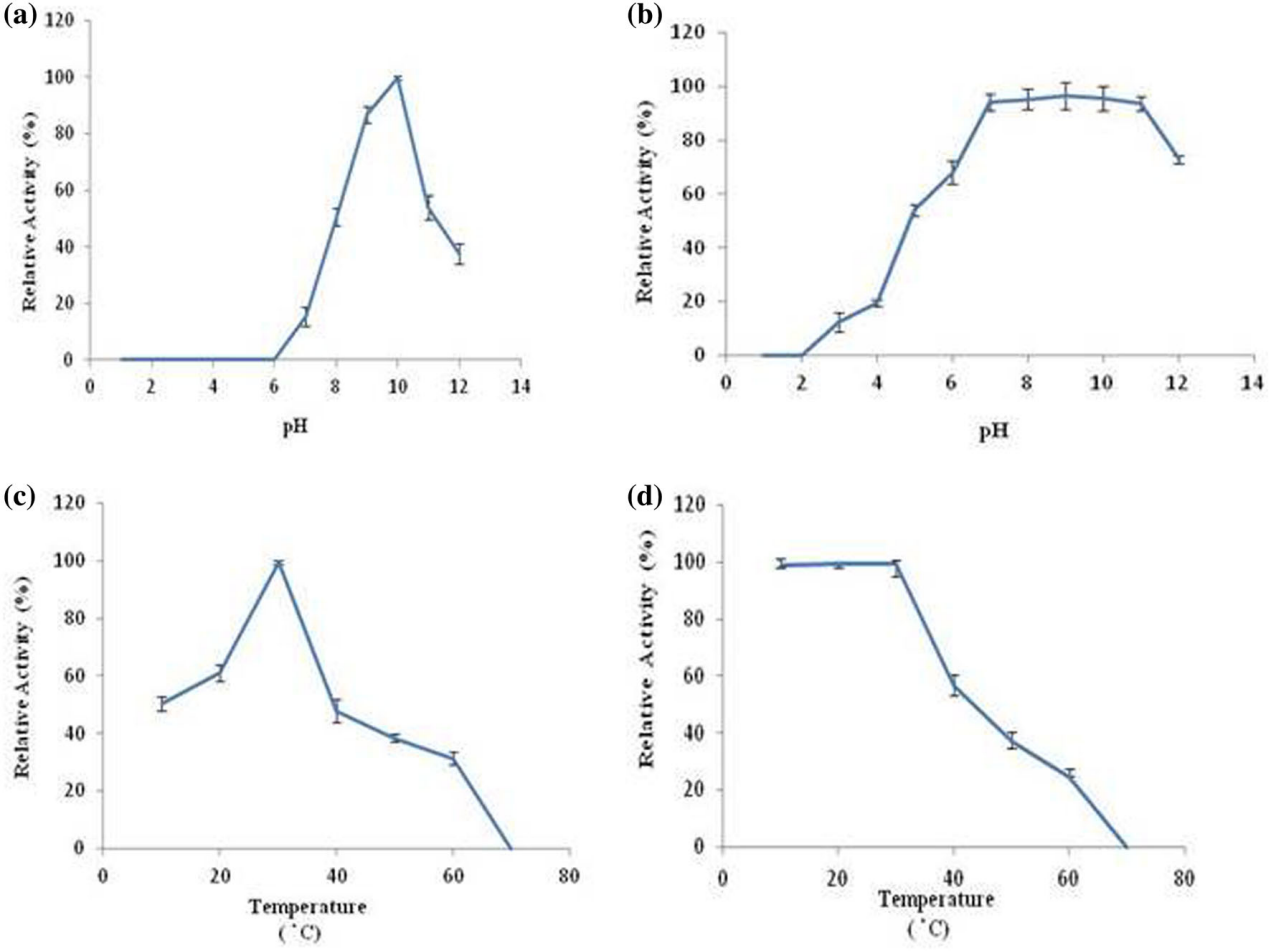
Effect of pH and temperature on the activity and stability of purified PG: **a** pH optima, **b** pH stability, **c** temperature optima, **d** temperature stability

The optimum pH for the purified polygalacturonase was found to be 10.0 and the enzyme was stable for a pH range, i.e., 7.0–11.0 (Fig. 2a, b) when exposed to buffers of various pH for 24 h, indicating its suitability for retting of natural fibers. Even at a high pH of 12.0, more than 70 % of the activity was retained. An alkaline PG with pH optima 10.0 has been reported from *Bacillus* sp. (Kapoor et al. 2000). The author could not find report of alkaline PG from fungal sources for comparison.

### Effect of temperature on the activity and stability of PG

Optimum temperature for the purified polygalacturonase activity was found to be 30 °C and the enzyme retained its maximum activity between 10 and 30 °C for 2 h (Fig. 2c, d). Approximately, 54 % of the activity was found at 40 °C which declined to 38.4 % at 50 °C (Fig. 2d). Optimum temperature of 34 °C has been reported for endo-PG from pathogenic fungus *Ustilago maydis* (Castruita-Domínguez et al. 2014). Similar temperature optimum of 30 °C has been reported for exo-PG from *Paecilomyces variotii* (Patil et al. 2012).

### Kinetic parameters

The apparent *Km* value for degradation of PGA by the purified enzyme was found to be 2.4 mg/mL (data not shown). Endo-PGs from *Penicillium frequentans* and *Saccharomyces cerevisiae* have *Km* values 2.7 and 4.2 mg/ml respectively **(**De Fatima Borin et al. 1996; Blanco et al. 1994 **)**. The catalytic rate constant (*k*_*cat*_) of purified PG was found to be 44 s^−1^. The *k*_*cat*_ values of 90 and 70 s^−1^ for endo-PGs from *Aspergillus japonicus* and *Fusarium moniliforme* have been reported (Semenova et al. 2002; Niture et al. 2001). Exo-PG from alkaliphilic *Bacillus* has *k*_*cat*_ value 22.2 s^−1^ (Kapoor et al. 2000).

### Effect of metal ions and protein inhibitors

The effect of different metal ions and protein inhibitors on the activity of the purified PG was attempted. The concentration of metal ions was kept 1 mM concentration in the reaction solution (Table 2). The metal ions namely Cu^2+^ and K^+^ were found to enhance the PG activity, while Ag^+^, Ca^2+^ and Hg^2+^ inhibited the enzyme activity. Metal ions, namely Mn^2+^, Na^+^, Zn^2+^ and protein inhibitor KMnO_4_ had no effect on PG activity.

**Table 2:** Effect of metal ions and protein inhibitors on polygalacturonase activity

S. no.	Metal ions (1.0 mM)	Relative activity (%)
	Control	100.0
1	Ag^+^	34.0
2	Ca^2+^	29.5
3	Co^2+^	95.0
4	Hg^+^	64.2
5	K^+^	135.5
6	Cu^2+^	163.0
7	Zn^2+^	95.0
8	Na^+^	100.0
9	Mn^2+^	95.0

S. no.	Protein inhibitors	Relative activity (%)
1	EDTA	85.7
2	Potassium permanganate	96.0
3	Potassium ferrocyanide	64.2

### Mode of action of purified PG

**Fig. 3:**
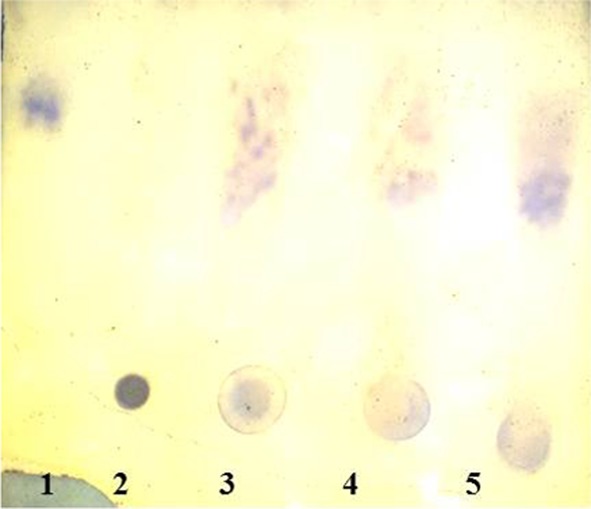
TLC of purified PG from *A. fumigatus* MTCC 2584. *Lane 1* Monogalacturonic acid, *Lane 2* Polygalacturonic acid, *Lane 3* Reaction after 15 min, *Lane 4* Reaction after 30 min, *Lane-5* Reaction after 45 min

The mode of action of purified PG was determined by TLC method as shown in Fig. 3. The reaction spots appeared between monogalacturonic acid and polygalacturonic acid spot, revealing the endo-type of reaction mechanism producing oligogalacturonates by random cleavage as manifested by spots visible between the monomer and the polymeric substrate. Endo-PGs of molecular weight 40.0 and 39.7 kDa have been reported from *F. graminearum* and *Phytophthora parasitica*, respectively (Ortega et al. 2014; Yan and Liou 2005).

### Application of purified PG in retting of *Crotalaria juncea* fibers

**Fig. 4:**
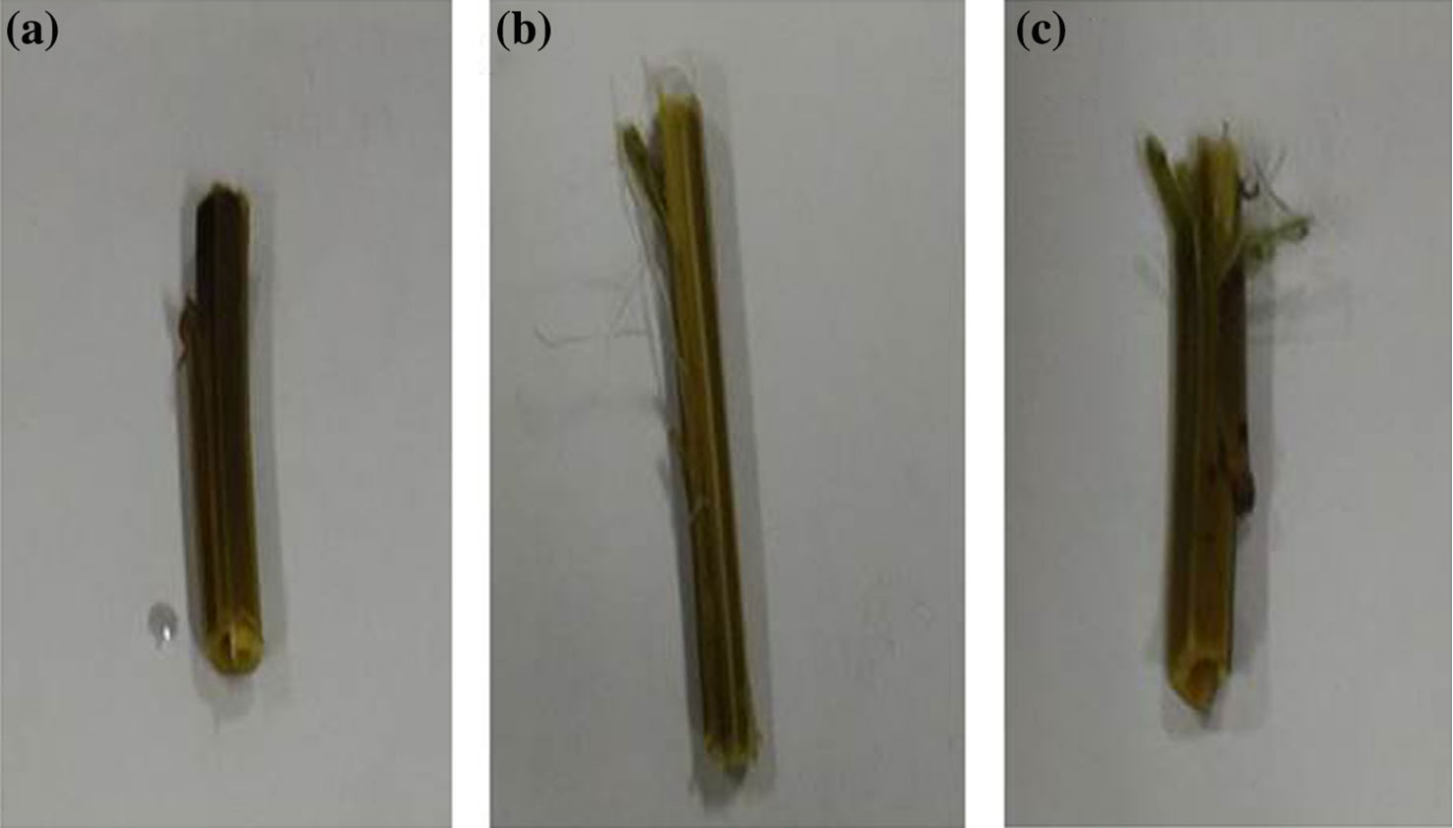
Retting of *Crotalaria juncea* fiber: **a** Control without enzyme, **b** with EDTA, **c** without EDTA by purified PG from *Aspergillus fumigatus* MTCC 2584

Microbial enzymes are extensively applied for retting of fibers including the pectinases (Yadav et al. 2016). The purified PG showed retting of locally available *Crotalaria juncea* natural fiber in the absence of EDTA (Fig. 4c). In general, acidic pectins and Ca^2+^ are located preferentially in the epidermal regions of *Linum usitatissimum* (flax) (Rihouey et al. 1995), contributing to the structural integrity of the stem and bast fibers. Therefore, chelators, such as EDTA, have the ability to remove Ca^2+^ and thus enhancing retting of flax (Henriksson et al. 1997, 1999). The variability in the pectin content along with Ca^2+^ in different fibers might influence the retting efficiency in the presence or absence of EDTA. Since EDTA was found to be slightly inhibitory to polygalacturonase activity, hence lower retting efficiency was observed in the presence of EDTA. The role of alkaline and thermostable polygalacturonase from *Bacillus* sp. MG-cp-2 in degumming of ramie (*Boehmeria nivea*) and sunn hemp (*Crotolaria juncia*) bast fiber has been reported (Kapoor et al. 2001). It has also been reported that a purified (endo) PGase from *Aspergillus niger* was capable of retting flax (Zhang et al. 2000).

## Conclusions

An endo-PG from *Aspergillus fumigatus* MTCC 2584 produced by solid-state fermentation was purified by acetone precipitation and gel-filtration chromatography resulting in 18.43-fold purification with specific activity of 38.9 IU/mg protein and 2.98 % yield. The purified PG has a relative molecular mass of approximately 43.0 kDa as revealed by SDS–PAGE and was alkaline in nature with pH optima of 10.0. The enzyme was found to be efficient in retting of natural fiber *Crotalaria juncea* in the absence of EDTA. To the best of our information, this is probably the first report of PG from *Aspergillus fumigatus*.
